# lncRNA OIP5-AS1 Suppresses Cell Proliferation and Invasion of Endometrial Cancer by Regulating PTEN/AKT via Sponging miR-200c-3p

**DOI:** 10.1155/2021/4861749

**Published:** 2021-07-28

**Authors:** Yun Liu, Xiaohui Cai, Yixuan Cai, Yue Chang

**Affiliations:** Department of Obstetrics and Gynecology, Beijing Friendship Hospital Affiliated to Capital Medical University, Beijing, China

## Abstract

**Background:**

Endometrial carcinoma (EC) is one of the major gynecologic malignancy cancers affecting females with dismal prognosis and high mortality around the world. Numerous studies have proven that an aberrant level of long noncoding RNAs is present in many endometrial cancer patients, while the underlying molecular mechanism remains unclear.

**Method:**

The expression levels of lncRNA OIP5-AS1, miR200c-3p, and PTEN were measured by a quantitative real-time polymerase chain reaction in endometrial cancer tissue and endometrial cancer cells. CCK8 assay, wound-healing assay, and cell colony formation were applied to evaluate cell proliferation, cell migration, and cell colony formation ability. Cell cycle and cell apoptosis were detected by flow cytometry. The interactions between OIP5-AS1, miR200c-3p, and PTEN were explored by luciferase activity.

**Results:**

In the present study, we demonstrated that long noncoding RNA OIP5-AS1 was significantly reduced in EC tissue compared with normal tissue. The lower expression level of OIP5-AS1 was also confirmed in four kinds of EC cell lines compared with the normal endometrial cell line. Gain- and loss-of-function of experiments indicated that upregulation of OIP5-AS1 could inhibit the proliferation, migration, and invasion of EC cells *in vitro*. Meanwhile, overexpression of OIP5-AS1 could also suppress the growth of tumor in the xenograft model. Moreover, further study revealed that miR-200c-3p could bind to OIP5-AS1, and the loss function of miR-200c-3p could reverse the elevated OIP5-AS1's inhibitory effect on the progression of EC. Furthermore, we found that downregulation of miR-200c-3p was inversely correlated with PTEN expression in EC cells. Reduced OIP5-AS1 could lead to the accumulation of miR-200c-3p, which could induce the upregulation of PTEN indirectly.

**Conclusion:**

Our study demonstrated a novel molecular mechanism that lncRNA OIP5-AS1 could modulate the progression of EC by combining competitively with miR-200c-3p to control the PTEN/AKT pathway in EC cells, which might supply important information for developing novel therapeutic strategies for EC patients.

## 1. Introduction

Endometrial cancer (EC), one of the deadliest gynecologic malignancies, is the fourth most common cancer type in women. Its dismal prognosis and increasing incidence make endometrial cancer a major cause of mortality in pre- and postmenopausal women worldwide [1]. The Cancer Genome Atlas has recently defined four molecular subtypes of endometrial cancer based on somatic mutations, copy number alterations, and microsatellite instability status [2], which proved that molecular alterations in EC might affect the development of EC, but it was a far from comprehensive molecular pathogenesis for us to develop targeted therapies. Therefore, investigating the molecular mechanisms underlying EC progression is imperative to supply important information for developing novel therapeutic strategies for EC patients.

Noncoding RNAs (ncRNAs) are a new class of transcripts encoded by genomes, but most of them are not translated into proteins. Although not translated, ncRNAs play key roles in cellular processes and pathogenesis [3, 4]. In particular, one subclass known as long noncoding RNAs (lncRNA) composed of more than 200 nucleotides in length have been demonstrated to be systemic regulators in multiple biologic processes, including tumorigenesis [5]. In previous studies, lncRNA HOTAIR has been demonstrated to reprogram the chromatin state to modulate breast cancer metastasis by altering the methylation of histone H3 lysine 27. Notably, it also has been defined as a critical element in endometrial cancer [6, 7]. Besides HOTAIR, a subset of lncRNAs including H9, MALAT1, and GAS5 have been proven to have an anomalous level in EC patient tissue, affecting the occurrence, development, and metabolism of endometrial cancer through different mechanisms [8–10]. Although only a few lncRNAs have been fully investigated, it is clear that lncRNAs provide the emerging opportunities and challenges of targeting lncRNA to diagnose and treat EC in the future [11].

OIP5 antisense transcript 1 (OIP5-AS1) is a conserved long noncoding RNA located on human chromosome 15q15.1 and was first identified to affect embryonic developmental defects in zebrafish in 2016 [12]. With the in-depth study of OIP5-AS1, increasing evidence has proven that OIP5-AS1 functions as an extremely important role in various carcinomas. Deng et al. demonstrated that OIP5-AS1 might function on the progression of lung adenocarcinoma via targeting miR-488. Meanwhile, Hu et al. published their study concerning OIP5-AS1, revealing that OIP5-AS1 could inhibit glioma cells' proliferation and migration through regulating the NOTCH pathway [13]. Interestingly, Jiang et al. conducted the integrative analysis of lncRNAs in 191 primary endometrial tumors using RNAseq and bioinformatics methods and found that OIP5-AS1 has a lower level in EC tissues compared with normal endometrium [14]. However, the correlation between OIP5-AS1 and EC remains uncertain.

In our current study, we investigated the biological role of OIP5-AS1 in EC progression. Our results showed that OIP5-AS1 was observably downregulated in EC cells. Meanwhile, gain- and loss-of-function experiments revealed that OIP5-AS1 could inhibit the proliferation and migration of EC cells in vitro and in vivo. Interestingly, we also found that OIP5-AS1 might directly associate with miRNA miR-200c-3p in EC cells. To investigate whether OIP5-AS1 impedes the progress via miR-200c-3p, we detected the level of PTEN, which was hypothesized as a potential target of miR-200C-3p using the starBase database. Furthermore, we confirmed the hypothesis for OIP5-AS1 and PTEN in endometrial cancer tissue. In conclusion, our data revealed that lncRNA OIP5-AS1 could suppress EC cells' proliferation and invasion through regulating PTEN pathways via sponging miR-200c-3p.

## 2. Materials and Methods

### 2.1. Specimen Collection

Eight endometrial tumor samples and corresponding normal endometrial tissues were collected from patients during surgery at the Department of Obstetrics and Gynecology, Beijing Friendship Hospital affiliated to Capital Medical University. Tissues were frozen at once in liquid nitrogen until needed. All these clinical materials were approved by the ethics committee of the Beijing Friendship Hospital (approval number: 18-2032). All patients signed consent forms. Eight pairs of tissue samples were applied for lncRNA OIP5-AS1 expression measurement.

### 2.2. Cell Culture

The endometrial cancer cell lines (HEC-1-A, RL-952, JEC, and KLE) and the normal cell line were obtained from the American Type Culture Collection (ATCC, Manassas VA, USA). HEC-1-A cells were cultured in McCoy's 5A (modified) Medium (Thermo, 16600082) with 10% fetal bovine serum (FBS, Thermo). RL-952 cells were cultured in DMEM/F-12 medium (GIBCO, 12400024) with 10% FBS. JEC and KLE cells were cultured in DMEM containing 10% FBS. All cell lines were maintained in a humidified incubator with 5% CO_2_ at 37°C. Endometrial cancer cell lines were used for constructing gain- or loss-of-function cell models in the present study.

### 2.3. Quantitative Real-Time PCR (qRT-PCR)

The total RNAs and miRNA were extracted from cells by using an RNAprep Pure Cell Kit (Tiangen, DP430). The experiment was performed according to the manufacturer's instructions. A PrimeScript™ RT Reagent Kit (Takara, RR047A, Japan) was applied in a reverse transcription assay. The relative level of miRNA and lncRNA was calculated using the SYBR Green qPCR Master Mix (Bimake, B21203, USA) on ABI Prism®7500 (ABI, USA). The data was assessed using the 2 − ΔΔCT. The primers used in the article were as follows: GAPDH, 5′-GGGAGCCAAAAGGGTCAT-3′ (forward) and 5′-GAGTCCTTCCACGATACCAA-3′ (reverse); OIP5-AS1, 5′-TGCGAAGATGGCGGAGTAAG-3′ (forward) and 5′-TAGTTCCTCTCCTCTGGCCG-3′ (reverse); and U6, 5′-CTTCGGCAGCACATATAC-3′ (forward) and 5′-GAACGCTTCACGAATTTGC-3′ (reverse).

### 2.4. Cell Viability Assay

The cells were seeded into the 96-well plates at a density of 1,000 cells per well after being transfected with RNAi or vector for 48 hours. After transfection, the cells were cultured for 12, 24, 48, and 72 hours. 10 *μ*l CCK8 medium was added to the well. The cells were continually cultured for 4 hours at 37°C. Then, absorbance was detected at the 450 nm wavelength.

### 2.5. Colony Formation Assay

The experiment was performed following the previous report [15]. After transfection for 48 hours, cells were resuspended in DMEM containing 0.35% FBS. The cells with limited density were plated in a 6-plate for almost two weeks. The cell colonies were stained with 0.1% (*w*/*v*) Crystal Violet (Sigma, USA) after being fixed using methanol.

### 2.6. Wound-Healing Assay

After transfection, the cells were subcultured in 6-well dishes. Wounds were created by a 10 *μ*l pipette tip. Cells were incubated overnight to yield confluent monolayers for wounding. The images were acquired at 0 h, 24 h, and 48 h after wounding. The distance migrated was measured by ImageJ. Wound healing rate = (distance (0 h) − distance (24 h/48 h))/distance (0 h).

### 2.7. Apoptosis Assay

Cell death was detected by an Annexin V-FITC/Propidium Iodide (PI) Apoptosis Detection Kit (Beyotime, Haimen, China). Cells transfected with related plasmids were resuspended in 500 *μ*l binding buffer with 5 *μ*l of Annexin V-FITC and 5 *μ*l of PI. The data were acquired by flow cytometry (BD FACSVerse cytometer) after 15 min incubation. The data were analyzed by FlowJo software.

### 2.8. Cell Cycle Analysis

Transfected cells were collected and washed with phosphate-buffered saline (PBS), then fixed using cold ethanol. The cells were incubated with RNase A (KeyGen Biotech, Nanjing, China) and PI (Sigma-Aldrich, St. Louis, USA). A FACS assay was performed through stained nuclei which were analyzed on a BD-FACSVerse flow cytometer (BD Biosciences, CA, USA).

### 2.9. Xenograft Model

The experiments in vivo were performed using 4-6-week-old BALB/C mice bought from the Shanghai Model Organisms Center. There were at least 6-8 mice in each group to establish the xenograft model. 5 × 10^6^HEC-1A cells with stably transfected OIP5-AS1 vector or Si-OIP5-AS1 were subcutaneously injected. Three weeks after the inoculation, tumor size, including length and width, was measured weekly. The tumor growth curve was drawn using the estimated data from the third to the seventh week. Mice were sacrificed at almost 30 days, and immunohistochemistry of Ki-67 and TUNEL was performed using nodules. All animal experiments were approved by the ethics committee.

### 2.10. Statistical Analysis

All the experiments were performed with at least three independent replicates. The results were presented as mean ± SDs, and graphs were created by GraphPad Prism 6.0. Statistical significance was evaluated by unpaired *t*-test or one-way ANOVA with Bonferroni's multiple comparison test. *P* < 0.05 was considered significant.

## 3. Results

### 3.1. lncRNA OIP5-AS1 Was Prominently Downregulated in EC Cells

**Figure 1:**
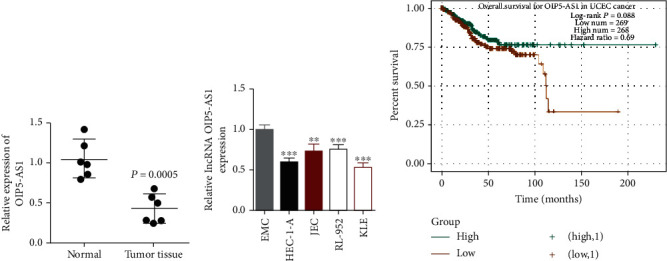
lncRNA OIP5-AS1 was poorly expressed in EC cells. (a) Relative expression of OIP5-AS1 in normal tissue and EC tissue from 8 patients. (b) The expression of OIP5-AS1 was significantly reduced in four EC cells compared with normal endometrial cells (ESC) by qRT-PCR. U6 was identified as an internal control. ^∗∗^*P* < 0.01 and ^∗∗∗^*P* < 0.001. (c) Result of the Kaplan-Meier method analysis manifested that the high expression group had longer overall survival in contrast to the high expression group. The data source was available from the online database starBase and TCGA.

To determine whether OIP5-AS1 has an aberrant presence in EC cells, we evaluated the level of OIP5- AS1 in human endometrial carcinoma tissues by real-time PCR. As shown in Figure 1(a), OIP5-AS1 is significantly upregulated in endometrial cancer compared to the normal endometrium. To confirm OIP5-AS1's expression in endometrial cancer cell lines, we also checked the level in four EC cell lines and the normal endometrial cell line (ESC) by qRT-PCR assay. The result indicated that the mean level of OIP5-AS1 in all EC cell lines (HEC-1A, RL-952, JEC, and KLE) was significantly lower compared with normal cells (*P* < 0.001, Figure 1(b)). Notably, it suggested that the abnormal presence of lncRNA OIP5-AS might be correlated with EC tumorigenesis. Using the mean relative expression of OIP5-AS1 as the threshold, we utilized a database online to identify the relationship between the OIP5-AS1 expression and the survival of EC patients [16]. The result of the Kaplan-Meier method analysis manifested that EC patients with higher OIP5-AS1 expression have a higher survival percent in contrast to those with a lower OIP5-AS1 expression, which suggested OIP5-AS1 expression was related to the prognosis for EC patients (Figure 1(c)).

### 3.2. Upregulation of lncRNA OIP5-AS1 Inhibited the Proliferation of EC Cell In Vitro

**Figure 2:**
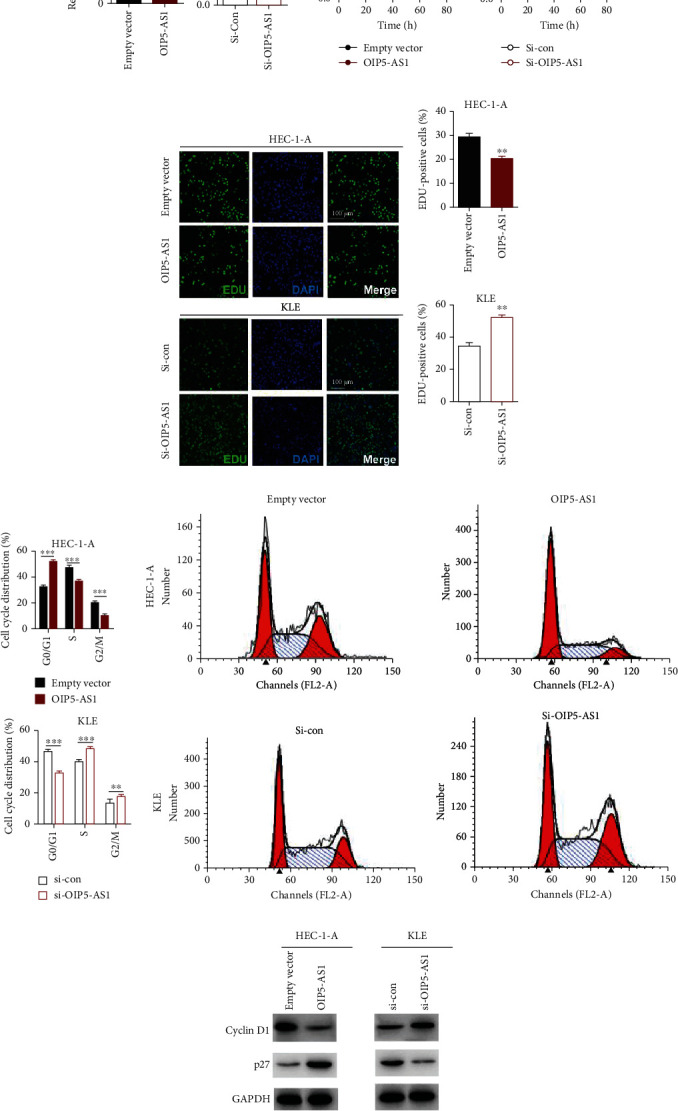
Upregulation of lncRNA OIP5-AS1 inhibited the proliferation of EC cell in vitro. (a) qRT-PCR was conducted to evaluate the expression of OIP5-AS1 in EC cell lines. After being transfected, OIP5-AS1 was significantly upregulated in the HEC-1A cell line compared with the empty vector group. Meanwhile, OIP5-AS1 was reduced in the KLE cell line by transfecting with the anti-OIP5-AS1 sequence. ^∗∗∗^*P* < 0.001. (b) CCK8 assay was used to measure cell vitality of HEC-1-A and KLE cells after transfection for 12, 24, 48, and 72 hours. (c) EDU staining was applied to label the DNA synthesis in HEC-1-A and KLE cells, which were transfected with OIP5-AS1 and its interfering RNA. Green represents EDU-positive cells; blue represents the cell nucleus. Scale bar 100 *μ*M. (d) Statistical result of EDU-positive cells. (e, f) Cell cycle was determined by flow cytometry. (g) Western blot analysis of cell cycle-related protein Cyclin D and p27 protein in the presence and absence of OIP5-AS1. Error bars represent the mean ± SEM of at least three independent experiments. ^∗∗∗^*P* < 0.001, ^∗∗^*P* < 0.01, and ^∗^*P* < 0.05.

To investigate the potential role of OIP5-AS1 in EC cells, we detected its regulation on the proliferation of EC cells by gain- and loss-of-function studies. lncRNA OIP5-AS1 was overexpressed in the HEC-1A cell line by cell transfection (OIP5-AS1 group) and was knocked down in the KLE cell line by transfecting the anti-OIP5-AS1 plasmid (Si-OIP5-AS1 group). In order to confirm the level of lncRNA OIP5-AS1 in the aforesaid cell lines, we performed qRT-PCR assay, and the result proved that OIP5-AS1 was significantly upregulated in HEC-1A compared to control cells (empty vector); meanwhile, it was downregulated in KLE in the Si-OIP5-AS1 group (*P* < 0.001, Figure 2(a)).

To examine whether OIP5-AS1 has functional impacts on the growth of endometrial cancer cells, the proliferation of stable EC cell lines with overexpression or knockdown of OIP5-AS1 was evaluated using CCK8. As shown in Figure 2(b), we found that upregulation of OIP5-AS1 resulted in significant decreases in EC cell viability compared to control cells. On the contrary, downregulation of OIP5-AS1 promoted the growth of EC cells. In order to identify OIP5-AS1's role on the EC cell proliferation via arresting cell cycle, we also stained the EC cells using EDU (5-ethynyl-2′-deoxyuridine), which could bind to newly synthesized DNA to measure DNA synthesis in living EC cells. From the data, we could learn that the number of EDU-positive EC cells is significantly reduced with overexpression of OIP5-AS1 (Figures 2(c) and 2(d)). Besides the EDU staining assay, we also conducted flow cytometry to detect the cell cycle status in EC cell lines. The result indicated that overexpression of OIP5-AS1 could make EC cells arrested at the G0/G1 stage through inhibiting the G1-to-S phase transformation (Figures 2(e)–2(f)). To explore the expression of cell cycle regulators, we detected the level of cell cycle-related protein Cyclin D and cell cycle inhibitor p27 via western blot. The data showed that the level of Cyclin D was reduced along with high-level p27 when overexpressing OIP5-AS1 in EC cells (Figure 2(g)). In conclusion, we found that OIP5-AS1 could suppress EC cells' proliferation via affecting cell cycle progression.

### 3.3. Overexpression of lncRNA OIP5-AS1 Could Restrain EC Cells' Tumorigenic Potential

**Figure 3:**
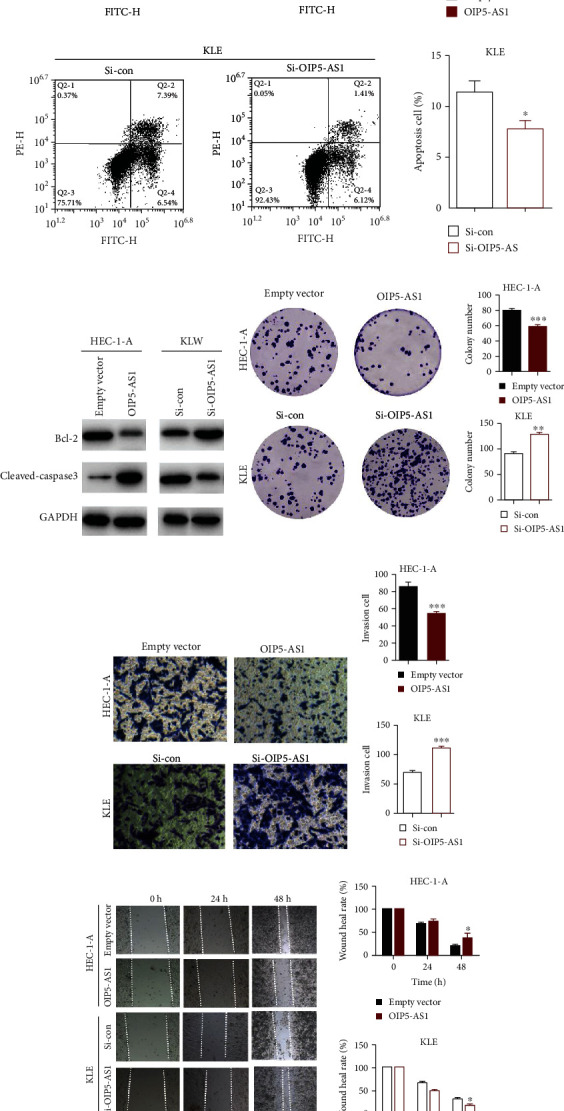
Overexpression of lncRNA OIP5-AS1 could promote the apoptosis and restrain EC cells' tumorigenic potential. (a) Measurement of apoptotic cells with OIP5-AS1 overexpression by FACS assay and calculated percentage of apoptotic cells. ^∗∗∗^*P* < 0.001 and ^∗^*P* < 0.05. (b) Western blot analysis of cell death-related protein Bcl-2 and cleaved-caspase3, and the internal control is GAPDH. (c) Colony formation assay detected cell proliferation in OIP5-AS1-upregulated HEC-1-A cells and OIP5-AS1-downregulated KLE cells. (d, e) Transwell assay to test the invasion ability of HEC-1-A and KLE by altering the level of OIP5-AS1. ^∗∗∗^*P* < 0.001. (f) Wound healing assay to identify the migration capacity of two EC cells after transfecting the OIP5-AS1 vector and Si-OIP5-AS1 for 48 hours. ^∗^*P* < 0.05.

In order to further demonstrate that OIP5-AS1 has adverse effects in endometrial cancer, we detected the cell death of EC cells by the PI/Annexin V flow cytometry assay and the TUNEL staining assay, which could determine different stages of apoptosis. Based on the flow cytometry analysis, we found that ectopic expression of OIP5-AS1 in the EC cells could promote the apoptosis progress, and downregulation of OIP5-AS1 could inhibit the process of cell death in EC cells (Figure 3(a)), which is consistent with the TUNEL assay's result (data not shown). Furthermore, we also confirmed the level of cell death-related protein Bcl-2 and cleaved-caspase3. As the western blot data showed, Bcl-2's level declined, and cleaved-caspase3 was observed to be elevated when upregulating OIP5-AS1 in EC cells. Meanwhile, the opposite phenotype could be observed when interfering with OIP5-AS1 in EC cells (Figure 3(b)). In total, these data demonstrate that high-level OIP5-AS1 could promote apoptosis of endometrial adenocarcinoma.

In the process of tumorigenesis, in addition to proliferation and apoptosis resistance, tumor cell invasion, migration, and tumorigenicity are also key characteristics of tumor formation and metastasis. In order to determine OIP5-AS1' function on cell migration and invasion, we conducted colony formation assay, wound-healing assay, and transwell assay (Figures 3(c)–3(f)). The result showed that upregulation of OIP5-AS1 also could restrain the EC cells' tumorigenic potential, which was embodied in the weak capability of invasion and migration.

### 3.4. Upregulation of OIP5-AS1 Could Suppress the Growth of Endometrial Tumor In Vivo

**Figure 4:**
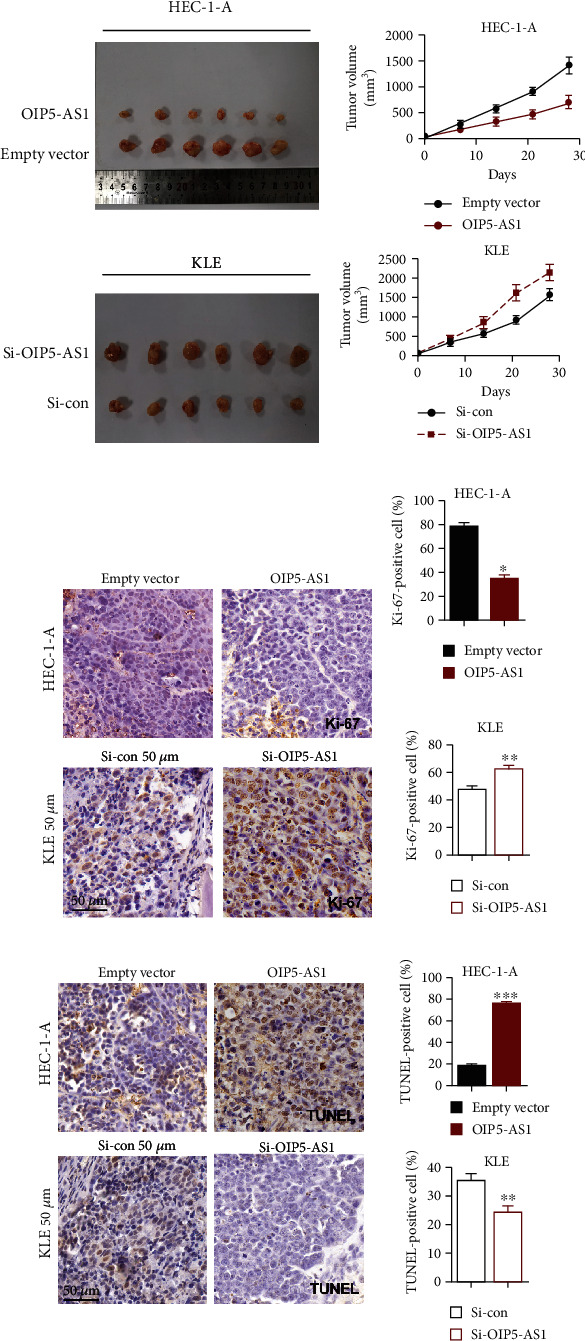
Upregulation of OIP5-AS1 could suppress the growth of endometrial tumors in vivo. (a) Representative images of EC xenografts formed by HEC-1-A and KLE cells while altering the OIP5-AS1 level. (b) Tumor growth cure represented the tumor's volume measured at 7 days, 14 days, 21 days, and 28 days. *N* = 6 each group. (c, d) Immunohistochemical staining for Ki-67 of tumor nodules and quantitative analysis. ^∗∗^*P* < 0.01 and ^∗^*P* < 0.05. (e) Immunohistochemical staining for TUNEL of tumor nodules and quantitative analysis. ^∗∗∗^*P* < 0.001 and ^∗∗^*P* < 0.01.

To further explore the impact of OIP5-AS1 in endometrial cancer in vivo, HEC-1A cells transfected with an empty vector and an OIP5-AS1 vector were subcutaneously injected in nude mice. As shown in Figures 4(a) and 4(b), upregulation of OIP5-AS1 significantly represses the growth of tumor size. Meanwhile, KLE cells with transfecting Si-OIP5-AS1 were also implanted into nude mice, and the result indicated that the size of tumor growth in the Si-OIP5-AS1 group was slightly smaller than that in the Si-control group, which suggested that downregulation of OIP5-AS1 accelerated the formation of xenografted tumors. To investigate the role of OIP5-AS1 in the proliferation and apoptosis of EC cells in vivo, we performed the immunohistochemical staining of Ki67 and TUNEL in xenografted tissues. The result revealed that the number of Ki67-positive cells was reduced with more TUNEL-positive cells in the OIP5-AS1 overexpression group, while the adverse result was observed in the OIP5-AS1 downregulation group (Figures 4(c)–4(f)). Taken together, all these data demonstrated that overexpression of OIP5-AS1 could inhibit the tumor progression of endometrial cancer in the xenograft model.

### 3.5. OIP5-AS1 Interacted with miR-200c-3p Directly and Modulated Its Level Negatively in EC Cells

**Figure 5:**
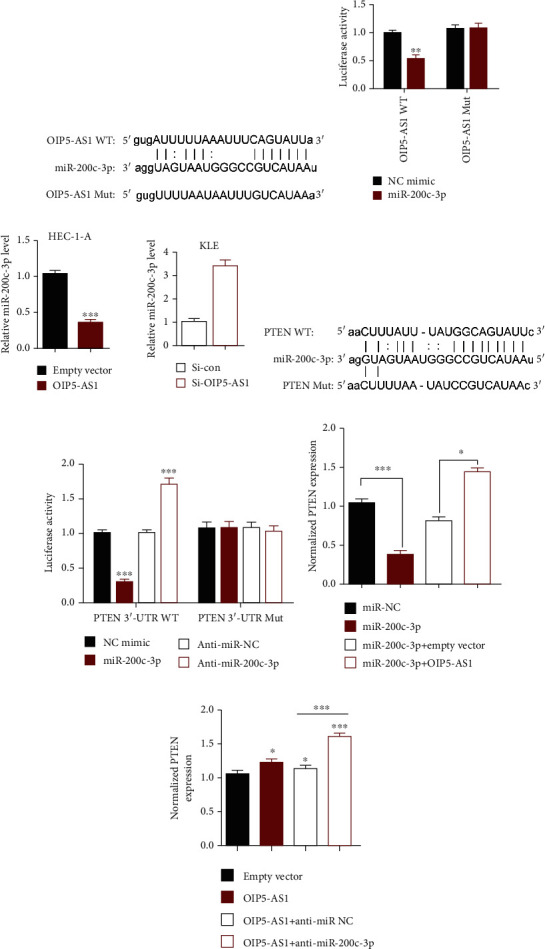
OIP5-AS1 could regulate PTEN's expression via sponging miR-200c-3p. (a) Predictive binding sequence of miR-200c-3p and OIP5-AS1 by starBase 3.0. (b) Dual-luciferase reporter assay showed that miR-200c-3p mimics reduced fluorescence intensity of OIP5-AS1-WT, while having no influence in OIP5-AS1-Mut. (c) The level of miR-200c-3p was detected using qPCR assay when altering OIP5-AS1. (d) Predictive binding sequence of miR-200c-3p and PTEN by starBase 3.0. (e) Dual-luciferase reporter assay showed that miR-200c-3p could affect PTEN-3′-UTR WT but not PTEN-3′-UTR Mut. (f, g) The transcriptional level of PTEN was measured using a qPCR assay while affecting miR-200c-3p and OIP5-AS1.

Increasing evidence has manifested that lncRNAs could exert their biological roles by acting as a molecular sponge or a competing endogenous RNA (ceRNA), which revealed the research mechanism that lncRNAs could regulate the accumulation of miRNA, thereby affecting the level of mRNA. To identify whether OIP5-AS1 repressed the process of endometrial cancer via acting as a ceRNA, we predicted its targets using online software starBase 2.0 then found that miR-200c-3p might be a potential partner of OIP5-AS1 (Figure 5(a)). Interestingly, previous work has indicated that the level of miR-200c-3p is higher in EC patients' tissue, while the underlying mechanism had not been revealed. To verify whether OIP5-AS1 could interact with miR-200c-3p, we conducted a dual-luciferase reporter assay that showed that miR-200c-3p could repress the luciferase activity in EC cells transfected with OIP5-AS1 3′UTR-Wt but have no influence in the OIP5-AS1 3′UTR mutant group (Figure 5(b)). Meanwhile, qRT-PCR showed that the upregulation of OIP5-AS1 could lead to reduced miR-200c-3p in EC cells (Figure 5(c)). In conclusion, OIP5-AS1 could sponge miR-200c-3p directly in EC cells.

### 3.6. OIP5-AS1 Could Regulate PTEN's Expression via Sponging miR-200c-3p

A growing body of evidence has indicated that the initiation and progression of endometrial cancer are controlled by different signaling pathways activated, including the AKT pathway [17, 18]. To find out which is the downstream gene of miR-200c-3p, we used a biological information tool online to predict miR-200c-3p's target and found that PTEN might be the candidate (Figure 5(d)). It is noticeable that PTEN has been reported to be one of the cancer suppressor genes involving in the AKT pathway. Thus, we proposed the hypothesis that miR-200c-3p might be related to PTEN in EC cells. To verify our thoughts, we tested the correlation between miR-200c-3p and PTEN by qRT-PCR. In addition, we could learn that there was a remarkable negative correlation between the level of miR-200c-3p and the expression of PTEN using bioinformatics tools. In order to confirm whether miR-200c-3p could combine with PTEN, we adopted the luciferase reporter assay and found that the luciferase activity of PTEN wide type (PTEN-WT) was repressed with upregulated miR-200c-3p (Figure 5(e)). Additionally, we confirm the mRNA level of PTEN when overexpressing miR-200c-3p; the result declared that overexpression of miR-200c-3p could inhibit PTEN's expression and coexpression of OIP5-AS1 could partially rescue the phenotype caused by upregulation of miR-200c-3p (Figure 5(f)). We also found that the transcriptional level of PTEN was elevated after transfecting OIP5-AS1 in EC cells. Besides it, the positive effect of OIP5-AS1 on PTEN could be enhanced by cotransfecting anti-miR-200c-3p (Figure 5(g)). Overall, we could make the conclusion that OIP5-AS1 could affect PTEN's expression by combining with miR-200c-3p.

### 3.7. OIP5-AS1 Acted as a ceRNA of PTEN by Sponging miR-200c-3p

**Figure 6:**
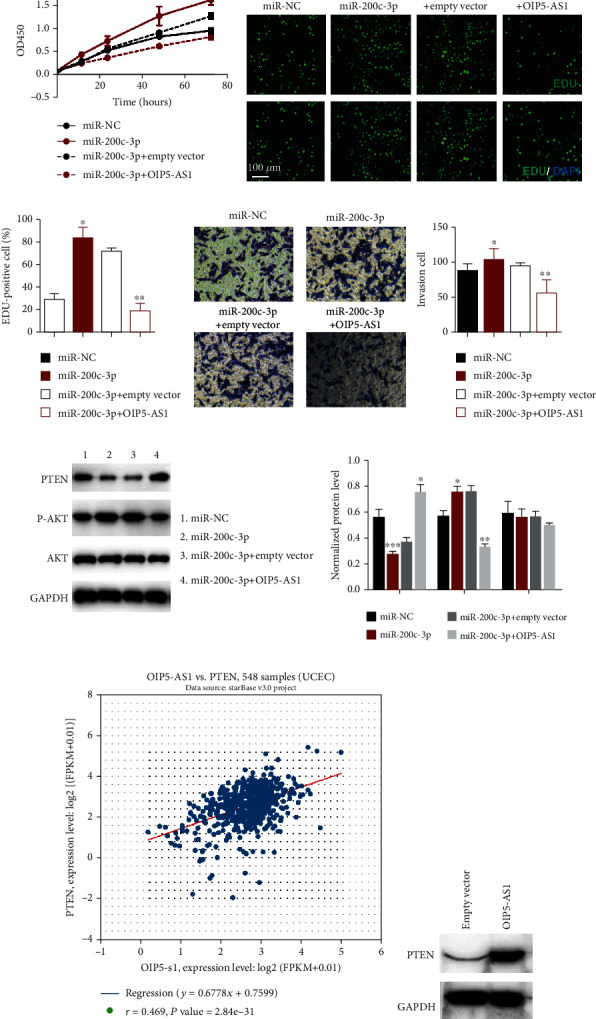
PTEN controls the progression of endometrial cancer by acting on a ceRNA of PTEN by sponging miR-200c-3p. (a) CCK8 assay was conducted to assess miR-200c-3p's influence on the HEC-1-A cell. (b, c) EDU staining images revealed that miR-200c-3p could promote proliferation, and coexpressing OIP5-AS1 could alleviate the effect caused by miR-200c-3p. (d) Transwell assay indicated that miR-200c-3p could facilitate tumor invasion. (e) Western blot of PTEN and AKT when miR-200c-3p and OIP5-AS1 are altered. (f) Data mining using the TCGA database exhibited a positive correlation between PTEN and OIP5-AS1. (g) Western blot assay proved the OIP5-AS1 overexpression upregulation of the level of PTEN in the HEC1A cell line.

To further explore whether OIP5-AS1's function on miR-200c-3p/PTEN was related to OIP5-AS1's antitumor effect in EC cells, we conducted the rescued experiment through cotransfecting OIP5-AS1 and miR-200c-3p in EC cells. After transfection, we firstly checked the proliferation of EC cells with the method of CCK8 and EDU staining. The results proved that coexpression of miR-200c-3p could reverse the effect on proliferation caused by upregulated OIP5-AS1 (Figures 6(a) and 6(b)). Then, we also detected the metastasis and invasion adopting transwell assay; the result proved that the overregulation of miR-200c-3p could promote the migration of the EC cell and the positive effects of miR-200c-3p were neutralized by the upregulation of OIP5-AS1 (Figure 6(c)). In addition, we also check the level of the PI3K pathway using western blot when altering OIP5-AS1 and miR-200c-3p (Figure 6(e)). The data showed that upregulation of PTEN caused by overexpression of OIP5-AS1 could be reversed when cotransfecting miR-200c-3p. Meanwhile, we also utilized the starBase online and TCGA to identify the relationship between OIP5-AS1 and PTEN. Results proved that OIP5-AS1 was positively correlated with PTEN expression (Figure 6(f)). Subsequently, a western blot assay was performed to identify the level of PTEN when overexpressing OIP5-AS1; results showed that upregulation of OIP5-AS1 could elevate the expression of PTEN (Figure 6(g)).

## 4. Discussion

**Figure 7:**
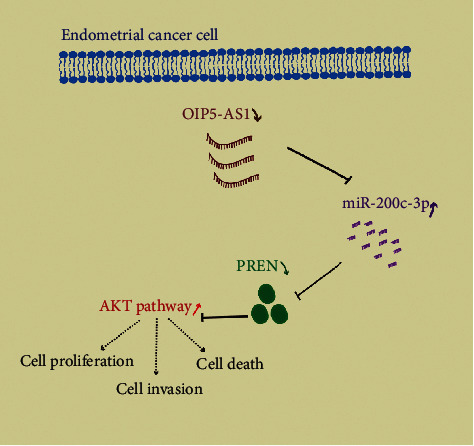
A schematic diagram depicts the molecular basis of OIP5-AS1/miR-200c-3p/PTEN in EC cells. In EC cells, the level of OIP5-AS1 was downregulated, which could promote the proliferation and invasion of EC *in vivo* and *in vitro*. Meanwhile, miR-200c-3p was the direct target of OIP5-AS1 and upregulated in EC cells, which was also related to the progression of EC. Notably, we found the miR-200c-3p could inhibit the expression of PTEN, which was the inhibitor of the AKT pathway. Taken together, we found a novel molecular mechanism that lncRNA OIP5-AS1 could act as a ceRNA to regulate PTEN by sponging the miR-200c-3p.

Endometrial cancer represents a huge problem among women worldwide due to its dismal prognosis and increasing incidence [19]. Given that depending on conventional chemotherapy or clinical surgery cannot solve the high mortality for advanced EC patients [20], the combination of multiple therapies provided a promising clinical strategy in the treatment of EC, such as immunotherapy and RNA therapy. Increasing evidence has proven that ncRNAs play an important role in the progression of tumors, while the latent mechanism is still unclear. Our study uncovered that overexpression of lncRNA OIP5-AS1 could impede the proliferation, migration, and invasion *in vitro* and *in vivo* for the first time. We found that OIP5-AS1's inhibitory function in EC relied on the miR-200c-3p, which was demonstrated to regulate PTEN's expression. Thus, we made the conclusion that OIP5-AS1 acted as a ceRNA to sponge miR-200c-3p in order to eliminate miR-200c-3p's effect on the PTEN/PI3K pathway (Figure 7).

miR-200c-3p belongs to the miR-200 family and has been reported to be a tumor-associated miRNA. Previous works have shown that miR-200c-3p takes part in diverse kinds of cancers, including colorectal cancer [21], prostate carcinoma [22], and renal cell carcinoma [23]. As for endometrial cancer, miR-200c-3p has been reported to be increased in EC tissue and could cooperate with lncRNA MALAT1, a classical long coding RNA related to endometrial tumorigenesis. The work uncovered that the miR-200c-3p/MALAT1 sponge could inhibit EMT in EEC, and it was identified to be enriched the most in EC tissue from EC patients in the previous study [24]. However, the certain downstream target of miR-200c-3p remains unclear [9]. In our study, we reveal the relationship between miR-200c-3p and PTEN in EC, which is a novel mechanism for endometrial tumorigenesis.

Multiple independent studies have revealed the fact that the PI3K/AKT pathway is a fundamental pathway that controls multiple biological processes [17]. A hyperactive PI3K/AKT pathway is found in different tumor tissues and is reported to promote the migration and growth of tumor cells [25]. Thus, finding a new component for eliminating the hyperactive PI3K/AKT pathway might be a good way to suppress the progression of tumor. The tumor suppressor PTEN was found to regulate the PI3K/AKT pathway via acting as a lipid phosphatase in EC for the first time in 2001 [26]. Along with the deepened study gradually, PTEN-positive and phosphorylated-Akt-negative expression has been identified as the predictor of survival for advanced endometrial carcinoma patients [26]. The abnormity of PTEN was found in several cancer types, which might be caused by gene deficiency, gene mutation, transcriptional inhibitor, and abnormal degradation by protease [25, 27, 28]. However, traditional drugs targeting PTEN especially small molecular drugs exhibited poorer druggability. As we know, RNA-targeted therapies represent a platform for drug discovery, including Onpattro approved in 2018. Unlike tumor immunology, RNAi therapy might reverse the level of cancer suppressor genes at the transcriptional level. Our study proved that the OIP5-AS1/miR-200c-3p sponge serves as a PTEN regulator, which has considerable diagnostic value and might be hopeful biomarkers for early detection and disease monitoring of EC. While the clinical application in our study needs further exploration in the future.

## 5. Conclusion

In the present study, we demonstrated that long noncoding RNA OIP5-AS1 was significantly reduced in EC cells, which suggested that OIP5-AS1 might play a role in EC tumorigenesis. Gain- and loss-of-function of experiments indicated that upregulation of OIP5-AS1 could inhibit the proliferation, migration, and invasion of EC cells *in vitro*. Meanwhile, overexpression of OIP5-AS1 could also suppress the growth of tumors in the xenograft model. Moreover, miR-200c-3p was predicted as a potential target of OIP5-AS1 by bioinformatics software. Further study revealed that miR-200c-3p could bind to OIP5-AS1, and the loss function of miR-200c-3p could reverse elevated OIP5-AS1's inhibitory effect on the progression of EC. Furthermore, we found that downregulation of miR-200c-3p was inversely correlated with PTEN expression in EC cells. Reduced OIP5-AS1 could lead to the accumulation of miR-200c-3p, which could induce the upregulation of PTEN indirectly.

## Data Availability

All data generated or analyzed during this study are included in this published article. Further details are available on request.

## Abbreviations

EC: Endometrial carcinoma
lncRNA: Long noncoding RNA
OIP5-AS1: OIP5 antisense RNA 1
PTEN: Phosphatase and tensin homolog
CCK8: Cell counting kit 8
qRT-PCR: Quantitative real-time polymerase chain reaction
UCEC: Uterine corpus endometrial carcinoma
ceRNA: Competing endogenous RNA.
